# EXAMINING AGEISM IN UNDERGRADUATE STUDENTS THROUGH DRAWING

**DOI:** 10.1093/geroni/igz038.184

**Published:** 2019-11-08

**Authors:** Katherina N Terhune, Aaron Guest, Tina M Kruger

**Affiliations:** 1 Northern Kentucky University, Highland Heights, Kentucky, United States; 2 University of Kentucky, Lexington, Kentucky, United States; 3 Indiana State University, Terre Haute, Indiana, United States

## Abstract

Substantial demographic shifts in the U.S. will result in the growth of the aging population and the need for qualified professionals entering the field of aging. Yet, these emerging professionals have limited exposure to aging curricula. It is vital that gerontologists recognize the ageism present in how older adults and the aging process are viewed, as this ultimately impacts how they are treated. Data from 1,609 undergraduate surveys from the multi-institution, multi-year Gerontological Literacy Network can assist in our understanding emerging professionals’ perspectives. Results indicate students incorrectly associate aging with loss of function and a reliance on assistive devices (canes 29.3% and glasses 19.6%), physical changes (balding 27.3% and wrinkles 30.3%), and greater likelihood of being male (32.5%). Aging is thought of as time passing (16.1%) and death (14.2%). Findings reveal a need to reframe students’ understanding of aging from that of decline to recognizing the strengths associated with age.

